# The role of perineal application of prophylactic negative-pressure wound therapy for prevention of wound-related complications after abdomino-perineal resection: a systematic review

**DOI:** 10.1007/s00384-020-03732-6

**Published:** 2020-09-04

**Authors:** Jeremy Meyer, Elin Roos, Ziad Abbassi, Christian Toso, Frédéric Ris, Nicolas C. Buchs

**Affiliations:** 1 Division of Digestive Surgery, University Hospitals of Geneva, Rue Gabrielle-Perret-Gentil 4, 1211 Genève 14, Switzerland; 2 Department of Global Public Health, Karolinska Institute, Stockholm, Sweden

**Keywords:** Abdomino-perineal amputation, Abdomino-perineal resection, Colorectal cancer, Rectal cancer, Infection, Perineal infection

## Abstract

**Background:**

Closed perineal wounds often fail to heal by primary intention after abdomino-perineal resection (APR) and are often complicated by surgical site infection (SSI) and/or wound dehiscence. Recent evidence showed encouraging results of prophylactic negative-pressure wound therapy (pNPWT) for prevention of wound-related complications in surgery. Our objective was to gather and discuss the early existing literature regarding the use of pNPWT to prevent wound-related complications on perineal wounds after APR.

**Methods:**

Medline, Embase, and Web of Science were searched for original publications and congress abstracts reporting the use of pNPWT after APR on closed perineal wounds.

**Results:**

Seven publications were included for analysis. Two publications reported significantly lower incidence of SSI in pNPWT patients than in controls with a risk reduction of about 25–30%. Two other publications described similar incidences of SSI between the two groups of patients but described SSI in pNPWT patients to be less severe. One study reported significantly lower incidence of wound dehiscence in pNPWT patients than in controls.

**Conclusion:**

The largest non-randomized studies investigating the effect of pNPWT on the prevention of wound-related complications after APR showed encouraging results in terms of reduction of SSI and wound dehiscence that deserve further investigation and confirmation.

**Electronic supplementary material:**

The online version of this article (10.1007/s00384-020-03732-6) contains supplementary material, which is available to authorized users.

## Introduction

Abdomino-perineal resection (APR) of the rectum consists of the ablation of the terminal colon, the rectum, the internal and external sphincters, and the confection of a terminal colostomy, as initially described by Miles [1]. APR is usually indicated for advanced adenocarcinomas of the lower third of the rectum (within 5 cm from the anal verge) and for recurrent squamous cell carcinoma of the rectum or anal margin after chemo-radiotherapy. Improvements to the techniques include neoadjuvant radio-chemotherapy for stages T 3–4 and/or radiologically node-positive adenocarcinomas, synchronous abdominal and perineal approaches, total mesorectum excision (TME, as introduced by Heald [2]), and minimally invasive techniques avoiding laparotomy for the abdominal approach.

Recently, emergence of sphincter-sparing procedures, such as partial and total intersphincteric resections for adenocarcinomas < 1 cm from the internal sphincter but sparing the external sphincter allowed reducing the indication for APR in favor of anterior resection. However, APR is still performed for rectal adenocarcinomas extending to the external sphincter, for incontinent patients, and for recurrent squamous cell carcinoma. Further, wider resections, such as extralevator abdomino-perineal excision (ELAPE [3]) removing the totality of levator ani muscles from their origin associated or not to multivisceral resection, are sometimes required in case of infiltration of levator ani muscles or surrounding organs, although indication for ELAPE is still debatable.

APR results in wide perineal defects. Usually, levator ani muscles are reapproximated using absorbable stiches. If a gap remains, the subcutaneous fat in the ischiorectal space or a synthetic or biological mesh can be used to fill the empty space [4]. In some cases, reconstruction using flaps, such as pedicled vertical rectus abdominis myocutaneous flap, local V-to-Y advancement flap (inferior gluteal artery perforator flap), and pedicled gracilis muscle flaps, is required [5, 6].

However, perineal wounds often fail to heal, notably due to preoperative radiotherapy side effects [7, 8], resulting in significant morbidity for patients, prolonged hospitalization, and increased costs for the healthcare system [9]. After failure to heal, a conventional negative-pressure wound therapy (NPWT) device (usually V.A.C .) is usually put in place to help healing by secondary intention.

Recently, NPWT preventively applied on closed wounds, also named prophylactic NPWT (pNPWT) or incisional NPWT (iNPWT), was reported to lower the risk of SSI after surgery in various surgical specialties, notably in gastrointestinal surgery [10–12]. Of interest, early publications reported encouraging results after APR, for which surgical wounds are more at risk of complications, but pooled evidence is lacking in that context.

The primary objective of the present systematic review was to gather and discuss the early existing literature regarding the use of pNPWT to prevent wound-related complications, notably SSI, on perineal wounds after APR.

## Methods

This systematic review was performed according to the Preferred Reporting Items for Systematic Reviews and Meta-Analyses (PRISMA) guidelines [13] (Table S1). MEDLINE, Embase, and Web of Science were searched from inception to 8 November 2019 for original studies written in English, Swedish, or French including patients who benefited from perineal application of pNPWT after APR. Search strategy is summarized in Table 1. Case series and conference abstracts were considered. Additional records were identified by manual search of the reference lists of the included publications. Secondary analyses of previously published papers and studies including patients < 18 years old were excluded. Studies were screened for inclusion by two authors (ER, JM). Discrepancies were solved by a third author (NCB). The systematic review and meta-analysis protocol was registered in the International Prospective Register of Ongoing Systematic Reviews (PROSPERO).

**Table 1:** Literature search strategy

Database	Search build	Occurrences
MEDLINE	((Negative-pressure[Title/Abstract]) OR (Negative pressure[Title/Abstract]) OR (Negative-pressure therapy[Title/Abstract]) OR (Negative pressure therapy[Title/Abstract]) OR (Negative-pressure wound therapy[Title/Abstract]) OR (Negative pressure wound therapy[Title/Abstract]) OR (Prophylactic closed-incision negative-pressure wound therapy[Title/Abstract]) OR (Prophylactic closed-incision negative pressure wound therapy[Title/Abstract]) OR (NPT[Title/Abstract]) OR (NPWT[Title/Abstract]) OR (pNPT[Title/Abstract]) OR (pNPWT[Title/Abstract])) AND ((perineal[Title/Abstract]) OR (perineum[Title/Abstract]) OR (abdominoperineal resection[Title/Abstract]) OR (abdomino-perineal resection[Title/Abstract]) OR (abdominoperineal excision[Title/Abstract]) OR (abdomino-perineal excision[Title/Abstract]) OR (APE[Title/Abstract]) OR (APR[Title/Abstract]))	77
EMBASE	('negative-pressure therapy':ti,ab,kw OR 'negative pressure therapy':ti,ab,kw OR 'negative-pressure wound therapy':ti,ab,kw OR 'negative pressure wound therapy':ti,ab,kw OR 'NPWT':ti,ab,kw) AND ('perineal':ti,ab,kw OR 'perineum':ti,ab,kw OR 'abdominoperineal resection':ti,ab,kw OR 'abdomino-perineal resection':ti,ab,kw OR 'abdominoperineal excision':ti,ab,kw OR 'abdomino-perineal excision':ti,ab,kw OR 'APE':ti,ab,kw OR 'APR':ti,ab,kw)	74
WEB OF SCIENCE	TI = (negative-pressure therapy OR negative pressure therapy OR negative-pressure wound therapy OR negative pressure wound therapy OR NPWT) ANDTI = (perineal OR perineum OR abdominoperineal resection OR abdomino-perineal resection OR abdominoperineal excision OR abdomino-perineal excision OR APE OR APR)	14

## Results

### Inclusion process

**Fig. 1:**
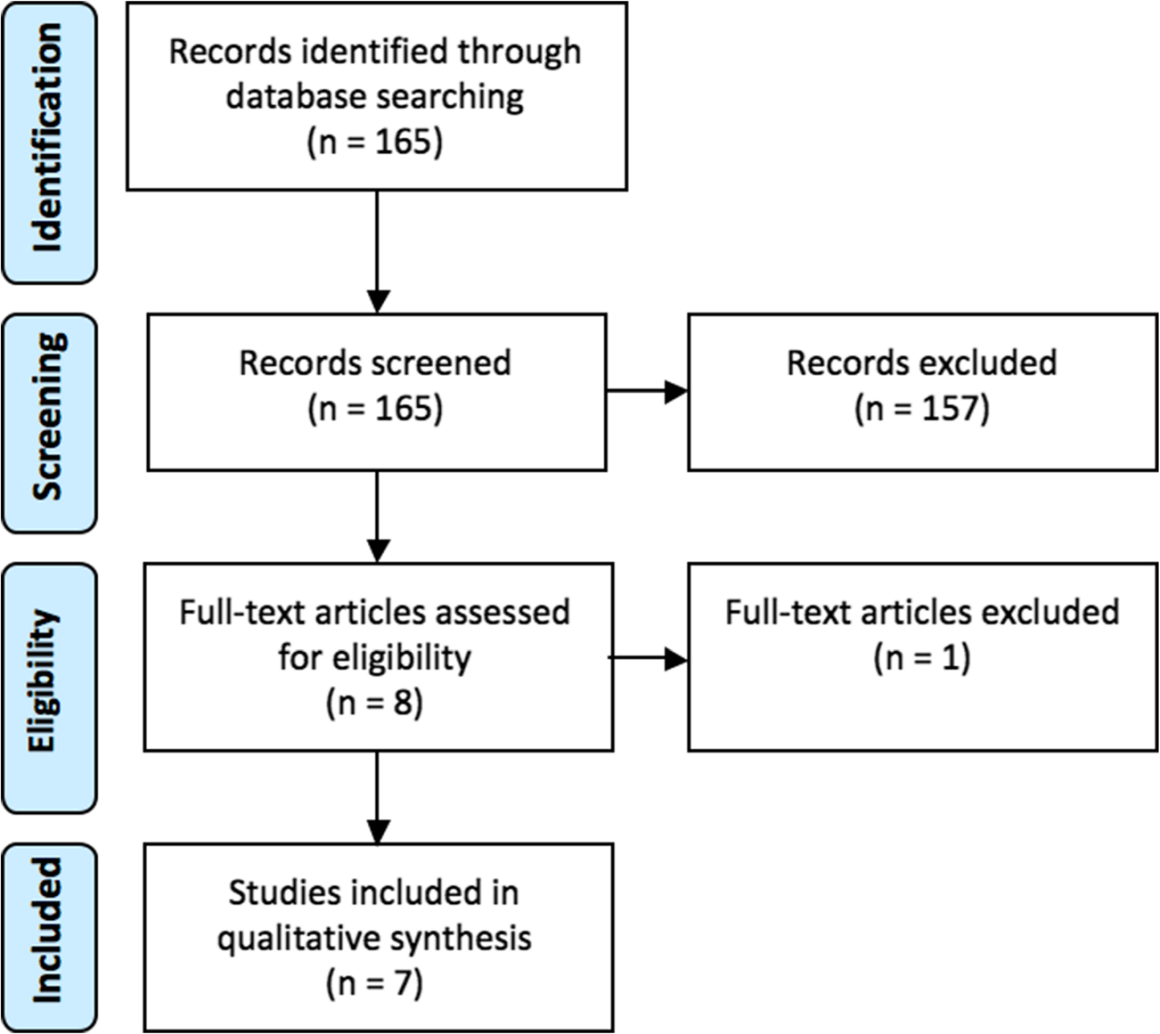
PRISMA flowchart

Seventy-seven publications were identified from MEDLINE, 74 from Embase and 14 from Web of Science. One hundred and fifty-seven records were excluded after abstract/title screening, and one after full-text screening, leaving seven publications [14–20] for definitive inclusion (Fig. 1).

### Characteristics of included studies

Included studies were composed of three congress abstracts [18–20] and four original publications [14–17]. All of them were recent, two being published in 2014 [14, 20], one in 2016 [15], two in 2017 [16, 17], and two in 2018 [18, 19]. All of them were cohort studies [14–20]. Six studies compared perineal wound outcomes between patients with pNPWT and those with conventional wound dressing [14–16, 18–20]. Control patients were historical in 3 studies [14–16] (Table 2).

**Table 2:** Characteristics of included studies

Authors	Year	Country	Type of publication	Type of study	Period
Chadi et al.	2014	Canada	Original publication	Retrospective cohort	2010–2012
Chung et al.	2014	USA	Congress abstract	Retrospective cohort	May 2009–September 2013
Rather et al.	2018	USA	Congress abstract	Retrospective cohort	-
Sumrien et al.	2016	UK	Original publication	Prospective cohort	November 2012–April 2015
Takahashi et al.	2018	Japan	Congress abstract	Cohort	-
Van der Walk et al.	2017	The Netherlands	Original publication	Prospective cohort with historical controls	January 2015–December 2015
Wiegering et al.	2017	Germany	Original publication	Cohort	-

All studies included patients undergoing APR [14–20] or pelvic exenteration [14, 17, 18]. Surgical indication was mainly rectal cancer [14–17], but two studies also included patients suffering from inflammatory bowel disease [14, 16]. Application of biological mesh to close the perineal defect and/or use of drain was poorly documented and varied among studies (Tables 3 and 4). In patients with application of pNPWT on closed perineum, the device used was the PREVENA incision management system (KCI, Acelity, San Antonio, USA) set at − 100 mmHg [17] or for 5 days [20] and the PICO single use negative pressure wound therapy system (Smith & Nephew, Hertfordshire, UK) set at − 80 mmHg for 4–8 days [16] or unknown commercial device set at − 125 mmHg for 5 days [14, 15].

**Table 3:** Characteristics of included studies in patients without prophylactic negative-pressure wound therapy

Authors	*n*	Indications for surgery	Preoperative treatment	Risk factors for SSI	Antibioprophylaxis	Surgery	Perineal closure	Mesh	Drain	Reconstruction	Follow-up
Chadi et al.	32	Rectal cancer, IBD, anal cancer	Immunosuppression, 4 (13%) Chemotherapy, 15 (47%) Radiotherapy, 16 (50%)	Smoking, 12 (38%) Diabetes, 4 (13%)	Yes	APR, 28 (88%) APR + proctocolectomy, 4 (13%)	Absorbable stiches for subcutaneous tissue and non-absorbable interrupted stiches for skin	No	No	No	30 days
Chung et al.	24	-	-	-	-	APR, 24 (100%)	-	-	-	-	-
Rather et al.	14	-	-	-	-	APR, 14 (100%)	-	-	-	-	≥ 14 (100
Sumrien et al.	25	Rectal cancer, 25 (100%)	Chemo-radiotherapy, 10 (25%)	-	-	APR, 25 (100	Absorbable stiches for subcutaneous tissue and skin	Biological	Yes	No	30 months (median)
Takahashi et al.	6	-	Chemo-radiotherapy, 6 (100%)	-	-	APR or pelvic exenteration, 6 (100%)	-	-	-	-	47.6-66.8 days (average)
Van der Walk et al.	10	Rectal cancer, 10 (100%)	Chemo-radiotherapy, 2 (20%) Radiotherapy, 3 (30%)	Smoking, 1 (10%) Cardiovascular comorbidity, 3 (30%)	Yes	APR, 10 (100%)	Various	Various	Various	Various	-
Wiegering et al.	0										

*IBD* inflammatory bowel disease, *APR* abdomino-perineal resection, *ELAP* extralevator abdomino-perineal resection, *SSI* surgical site infection. Numbers represent the number of patients (and the proportion)

**Table 4:** Characteristics of included studies in patients with prophylactic negative-pressure wound therapy

Authors	*n*	Indications for surgery	Preoperative treatment	Risk factors for SSI	Antibioprophylaxis	Surgery	Perineal closure	Mesh	Drain	Reconstruction	Follow-up
Chadi et al.	27	Rectal cancer, IBD	Immunosuppression, 6 (22%) Chemotherapy, 17 (63%) Radiotherapy, 16 (59%)	Smoking, 16 (59%) Diabetes, 3 (11%)	Yes	APR, 20 (74%) APR + proctocolectomy, 4 (15%) Pelvic exenteration, 3 (11%)	Absorbable stiches for subcutaneous tissue and non-absorbable interrupted stiches for skin	No	No	No	30 days
Chung et al.	22	-	-	-	-	APR, 22 (100%)	-	-	-	-	-
Rather et al.	16	-	-	-	-	APR, 16 (100%)	-	-	-	-	≥ 16 (100
Sumrien et al.	32	Rectal cancer, 32 (100%)	Chemo-radiotherapy, 13 (41%)	-	-	ELAPE, 32 (100%)	Absorbable stiches for subcutaneous tissue and skin	Biological	Yes	No	30 months (median)
Takahashi et al.	5	-	Chemo-radiotherapy, 5 (100%)	-	-	APR or pelvic exenteration, 5 (100%)	-	-	-	-	47.6-66.8 days (average)
Van der Walk et al.	10	Rectal cancer, 10 (100%) + IBD, 2 (20%)	Chemo-radiotherapy, 4 (40%)	Smoking, 2 (20%) Cardiovascular comorbidity, 5 (50%)	Yes	APR, 10 (100%)	Various	Various	Various	Various	-
Wiegering et al.	6	Rectal cancer, 5 (83%) Rectal NET, 1 (17%)	Chemo-radiotherapy, 6 (100%)	-	-	APR, 5 (83%) Pelvic exenteration, 1 (17%)	Non-absorbable interrupted stiches	-	-	VRAM flap, 1 (17%)	-

*IBD* inflammatory bowel disease, *APR* abdomino-perineal resection, *ELAP* extralevator abdomino-perineal resection, *SSI* surgical site infection. Numbers represent the number of patients (and the proportion)

### Perineal wound complications

Wiegering et al. reported one wound dehiscence (16.7% of patients) occurring after 8 days and requiring V.A.C. therapy for secondary healing [17].

Chadi et al. compared 27 patients with pNPWT with 32 patients with conventional wound dressing and found pNPWT patients to have significantly less SSI than control patients (14.8% versus 40.6%, *p* = 0.04). The incidence of intra-abdominal abscess (7.4% versus 3.1%, *p* = 0.59) or of emergency department visit (0% versus 6.2%, *p* = 0.18) did not differ between the two groups [14]. Chung et al. also reported significantly lower incidence of SSI among patients with pNPWT than in controls (9.1% versus 41.7%, *p* = 0.012) [20]. Rather et al., however, found similar incidence of SSI between patients with and without pNPWT (50% versus 64.3%) but described these infectious complications to be “less severe” in pNPWT patients. Noteworthy, 18.8% of pNPWT patients and 50% of control patients required V.A.C. therapy of the perineal wound for secondary healing. One patient who did not benefited from pNPWT required reoperation [19]. Van der Walk et al. reported similar incidences of SSI between pNPWT and control patients (70% versus 60%, *p* value not communicated). One patient from the conventional wound dressing group required reintervention [16].

Sumrien et al. reported significantly lower incidence of wound dehiscence in pNPWT patients than in controls (40% versus 9.4%, *p* = 0.01). Takahashi et al. observed a similar trend (0% versus 50%, *p* value not communicated) (Table 5).

**Table 5:** Reported perineal wound-related complications

pNPWT									Controls							
Authors	Patients, *n*	SSI, n (%)	Wound dehiscence, *n* (%)	Intra-abdominal abscess, *n* (%)	ED visit, *n* (%)	V.A.C., *n* (%)	Reoperation, *n* (%)	Length of stay (days)	Patients, *n*	SSI, n (%)	Wound dehiscence, *n* (%)	Intra-abdominal abscess, *n* (%)	ED visit, *n* (%)	V.A.C., *n* (%)	Reoperation, *n* (%)	Length of stay (days
Chadi et al.	27	4 (14.8%)	-	2 (7.4%)	0 (0%)	-	-	11**	32	13 (40.6%)	-	1 (3.1%)	2 (6.2%)	-	-	8**
Chung et al.	22	2 (9.1%)	-	-	-	-	-		24	10 (41.7%)	-	-	-	-	-	
Rather et al.	16	8 (50%)	-	-	0 (0%)	3 (18.8%)	0 (0%)		14	9 (64.3%)	-	-	-	7 (50%)	1 (7.1%)	
Sumrien et al.	32	-	3 (9.4%)	-	-	-	-		25	-	10 (40%)	-	-	-	-	
Takahashi et al.	5	-	0 (0%)	-	-	-	-	47.6***	6	-	3 (50%)	-	-	-	-	66.8***
Van der Walk et al.	10	7 (70%)	-	-	-	-	0 (0%)		10	6 (60%)*	-	-	-	-	1 (10%)	
Wiegering et al.	6	-	1 (16.7%)	-	-	1 (16.7%)	-		-	-	-	-	-	-	-	

*ED* emergency department, *SSI* surgical site infection, *V.A.C.* negative-pressure wound therapy on an open wound

*Number extracted from the text and not the table

**Median

***Average

## Discussion

In the present systematic review, we have included seven studies investigating the effect of pNPWT on the prevention of perineal wound complications after APR.

Two publications reported significantly lower incidence of SSI in pNPWT patients than in controls with a risk reduction of about 25–30% [14, 20], therefore showing encouraging results in favor of perineal pNPWT. Two other publications described similar incidence of SSI between the two groups of patients but described SSI in pNPWT patients to be less severe [19], or the authors believed that pNPWT could accelerate wound healing [16]. Further, one study reported significantly lower incidence of wound dehiscence in pNPWT patients than in controls (40% versus 9.4%, *p* = 0.01) [15].

We note that these studies were pilot studies, which presented several limitations. These were heterogeneous in designs, patients’ populations (with potential differences in terms of risk factors for wound-related complications), definition of controls (mainly historical), surgical procedures, pNPWT procedures (in terms of device, negative pressure applied, and duration of therapy—of note, several studies reported dysfunction of the device(s) requiring replacement and/or discontinuation of therapy [15, 16]), and reported outcomes (SSI, wound dehiscence, intra-abdominal abscess, emergency department visit, negative-pressure therapy for secondary healing, reoperation) and had small sample sizes, which constitute important limitations to their interpretation and prevent any meta-analysis of the actual literature in the field. However, we note that studies reporting a significant effect of pNPWT on the prevention of SSI and/or wound dehiscence were the studies with the largest sample sizes. Therefore, it might be likely that the absence of a significant effect observed in smaller studies might result from a type II error (lack of statistical power).

We believe that prevention of wound-related complications after APR is of crucial importance. For instance, these complications might increase length of stay with the subsequent risks of thrombo-embolic complications and nosocomial infections in these vulnerable patients and might also postpone administration of adjuvant therapy. We think that perineal application of pNPWT for the prevention of wound-related complications after ARP deserves further investigation, for example, with a large-enough randomized controlled trial, as the existing high incidence of perineal wound complications might allow to obtain important benefits even with a small effect of that therapy.

## Conclusion

The largest non-randomized studies investigating the effect of pNPWT on the prevention of wound-related complications after APR show encouraging results in terms of reduction of SSI and wound dehiscence that deserve further investigation and confirmation.

## Electronic supplementary material

ESM 1 (DOC 62 kb)
